# How Socio-economic Inequalities Cluster People with Diabetes in Malaysia: Geographic Evaluation of Area Disparities Using a Non-parameterized Unsupervised Learning Method

**DOI:** 10.1007/s44197-023-00185-2

**Published:** 2024-02-05

**Authors:** Kurubaran Ganasegeran, Mohd Rizal Abdul Manaf, Nazarudin Safian, Lance A. Waller, Feisul Idzwan Mustapha, Khairul Nizam Abdul Maulud, Muhammad Faid Mohd Rizal

**Affiliations:** 1 Department of Public Health Medicine, Faculty of Medicine, Universiti Kebangsaan Malaysia, 56000 Kuala Lumpur, Malaysia; 2 Clinical Research Center, Seberang Jaya Hospital, Ministry of Health Malaysia, 13700 George Town, Penang Malaysia; 3 Department of Biostatistics and Bioinformatics, Rollins School of Public Health, Emory University, Atlanta, GA 30322 USA; 4 Public Health Division, Perak State Health Department, Ministry of Health Malaysia, 30000 Ipoh, Perak Malaysia; 5 Earth Observation Centre (EOC), Institute of Climate Change, Universiti Kebangsaan Malaysia, 43600 Bangi, Selangor Darul Ehsan Malaysia; 6 Department of Civil Engineering, Faculty of Engineering and Built Environment, Universiti Kebangsaan Malaysia, 43600 Bangi, Selangor Darul Ehsan Malaysia

**Keywords:** Epidemiology, Cluster analysis, Socio-economic inequalities, Social determinants of health, Population indicators, Public health

## Abstract

Accurate assessments of epidemiological associations between health outcomes and routinely observed proximal and distal determinants of health are fundamental for the execution of effective public health interventions and policies. Methods to couple big public health data with modern statistical techniques offer greater granularity for describing and understanding data quality, disease distributions, and potential predictive connections between population-level indicators with areal-based health outcomes. This study applied clustering techniques to explore patterns of diabetes burden correlated with local socio-economic inequalities in Malaysia, with a goal of better understanding the factors influencing the collation of these clusters. Through multi-modal secondary data sources, district-wise diabetes crude rates from 271,553 individuals with diabetes sampled from 914 primary care clinics throughout Malaysia were computed. Unsupervised machine learning methods using hierarchical clustering to a set of 144 administrative districts was applied. Differences in characteristics of the areas were evaluated using multivariate non-parametric test statistics. Five statistically significant clusters were identified, each reflecting different levels of diabetes burden at the local level, each with contrasting patterns observed under the influence of population-level characteristics. The hierarchical clustering analysis that grouped local diabetes areas with varying socio-economic, demographic, and geographic characteristics offer opportunities to local public health to implement targeted interventions in an attempt to control the local diabetes burden.

**Supplementary Information:**

The online version contains supplementary material available at 10.1007/s44197-023-00185-2.

## Introduction

Human settlements have been known to affect population’s health; communities residing in socio-economically deprived areas are vulnerable to poor health outcomes due to the lack of basic infrastructural and social needs (e.g., accessibility and affordability to healthy food, coverage of health or exercise facilities, housing structure) in contrast to communities living in rich neighborhoods [1]. Public health researchers, epidemiologists, and policy advocates were convinced that wealth and poverty are effective predictors of health outcomes and life expectancy [1]. Higher income was associated with longer life expectancy compared to that of low-income individuals, and these differences increased over time [1]. These observed differences in longevity between income groups narrowed in some areas and widened in others, a finding that accelerated speculation that differences in life expectancies and health outcomes correlated with the intersection of people’s health behaviors and local neighborhood’s living circumstances [1].

Health disparities between and within local neighborhood areas are not only caused by inequitable access to health services but are also associated with geographic distributions of poverty and structural social determinants of health in which communities reside, play, and work [2]. In assessing small-area patterns of disadvantage and diabetes patterns, one study from Scandinavia found persistent connections between socio-economic disadvantage and diabetes prevalence across local municipalities [3]. In addition, individual and areal-level socio-economic indicators were found to increase the likelihood of diabetes incidence among populations in Saskatchewan, and the variability was strikingly influenced by the gradients of socio-economic scores between urban and rural areas [4]. Among diabetes adults from the USA, it was found that those who lived in socio-economically deprived areas had significantly higher rates of complications and emergency department visits when compared to those residing in non-deprived areas [5].

The above reported circumstances postulate a theory behind small-area social mechanisms, i.e., processes involving the social environment around neighborhoods fundamentally shaping the incidence and prevalence of observed health inequalities [6]. Understanding how these small-area health inequalities occur via macro (neighborhood) and micro (individual) roles within local social mechanisms through causal, mediation, or pathway processes motivates researchers to determine the strength of associations and plausibility of such interactions [6]. These social mechanisms are weighted geographically, and drive interactions between socio-economic inequalities and health outcomes across geographical areas providing evidence of specific needs for systematic local interventions and monitoring, according to observed gradients of disparities across populations [4].

The application of modern econometrics and related measures (e.g., median household income, Gini coefficient and other measures of income inequality, and incidence of poverty) nested within geographical epidemiology and spatial statistics reveals promising features to motivate and advance timely public health policy interventions [7]. However, the rise of multi-modal approaches from different settings and theories underlying econometric metrics within the local environments of social mechanisms reveal variations in associations across different settings, sometimes challenging the development of clearly defined, real-life interpretations [6, 8], a consequence that arises from the utilization of multi-sectoral secondary data sources [2].

Conventional public health approaches illustrate variations of health indicators or deprivation profiles through average scores or ranks; these areal scores are subsequently grouped by percentiles or deciles, which are subjected to misclassification, sometimes causing difficulties in capturing the true scenario of local disease burden areas [9, 10]. To synchronize these heterogeneous variations, epidemiologists grouped residential areas or neighborhoods with similar socio-economic and demographic attributes for ease of interpretation and focusing targeted interventions [10]. This rational could be realized by applying novel statistical clustering methods via unsupervised machine learning methods (i.e., learning from patterns of data) to collate geographical areas experiencing similar observed health needs and challenges. Advanced modern epidemiological and statistical partitioning (e.g., *k*-means clustering) or hierarchical clustering approaches offer opportunities to “cluster” or group areas with similar exposures and health outcomes via “branching” in dendrogram synthesis [11]. In recent times, hierarchical agglomerative clustering approaches have set preference to spatial statisticians and epidemiologists to allow clustering of areas to occur “naturally” within the set of data patterns, rather than analysts a priori determining the fixed number of clusters as in partitioning methods, which could bias the results [10, 11]. Hierarchical clustering approaches were preferred in applications to chronic health conditions (e.g., diabetes) because areal-level spatial patterns of population characteristics that accelerate disease risk (e.g., poverty) are unlikely to change in short time periods unlike infectious disease epidemics [12]. It should be noted that “clustering” of similar observations (outcomes and/or covariates) differs from geographical “clustering” of observations based on combining information of ‘nearest neighbors’ and/or parametric functions of distance [10]. As clustered domain rates might be skewed at the population level, non-parameterized tests are used in data science approaches to understand the characteristics that influence the synthesis of areal-level clusters [10].

This study applied hierarchical clustering (of similar observations) techniques to explore patterns of diabetes burden correlated with socio-economic inequalities in Malaysia, a country with the highest diabetes burden within the Western Pacific region [13]. Areas with similar characteristics of socio-economic inequalities among people with diabetes were clustered. The patterns on how these clusters differed according to their geographic, demographic, and population characteristics were explored.

## Methods

### Design, Setting, and Population

This was an ecological study involving 271,553 active type 2 diabetes cases aged 20 years and above captured between 2016 and 2020 across 914 primary care clinics in Malaysia.

### Data Sources and Processing

The source data of the main outcome variable includes the retrieval of active type 2 diabetes cases captured from all primary care clinics in Malaysia, officially registered in the National Diabetes Registry of Malaysia (NDR) [14, 15]. The NDR collects basic demographic, clinical, and outcomes information of diabetes patients registered from the participating primary care clinics (located within each district) serviced by the Ministry of Health Malaysia [14]. Data reporting total area size in square kilometers for each administrative district were obtained from the Department of Survey and Mapping Malaysia [16]. Geographic data of state boundaries and administrative districts were obtained from the Malaysia-Subnational Administrative Districts Data, United Nations Office for Coordination of Humanitarian Affairs [17]. Demographic data and population-level indicators at the administrative district level were obtained from the Malaysian Census 2020 [18]. Type 2 diabetes crude rates per 100,000 population were computed for all 144 administrative districts in Malaysia. The shapefiles for each data layer were spatially joined to curate the full set of attribute data.

### Cartography Development

To spatially visualize local areal-level diabetes rates and socio-economic inequalities (median household income, Gini coefficient, and incidence of poverty), bivariate choropleth maps were built with *n*^*2*^ classes (3 classes each for each variable yielding a total of 9 classes) to map the combinations of attribute variables most influencing the concentration of diabetes rates weighted geographically across the country. Using Quantum GIS (QGIS), shapefiles of state boundaries and administrative districts were layered and spatially joined to attribute data across layers. All bivariate 3 × 3 choropleth maps were built through the graduated function in “Symbology” using Natural Jenks optimization in QGIS, version 3.22 Bia**ł**owieza [19] (Plugins: Bivariate Legend) software. Results by tertile groups were displayed, that is 33% of the districts fall in each of the “low,” “medium,” and “high” categories. Next, this study sought to understand how areas cluster together, and what demographic, geographic, and population-level characteristics define and influence these clusters of diabetes.

### Statistical Analysis

This study utilized an unsupervised hierarchical agglomerative (bottom-up approach) clustering algorithm, as defined below [20–22]. The algorithm computes the Euclidean distance between each administrative district and compares average rates of diabetes correlated with socio-economic inequalities (median household income, Gini coefficient, incidence of poverty). The algorithm identifies two administrative districts experiencing the lowest distance score and links them into a cluster. This process is repeated until one cluster contains all the administrative districts.

#### Mathematical Algorithm Development

If **a** and **b** are allowed as two vectors of observations from each small area, with a length of *p* for each vector (where *p* denotes the number of small areas), a functional distance measure can be developed based on a Minkowski distance measure defined in vector space *R*^*p*^ [23, 24]:

1 $$D_{Minkowski} \,\left( {{\bf{a}},{\bf{b}}} \right) = \left[ {\sum_{i = 1}^p {\left| {a_i - b_i } \right|^r } } \right]^\frac{1}{r} ,$$

where *a*_*i*_ and *b*_*i*_ indicate the *i*th element for observations of vectors **a** and **b,** respectively. The distance advocated by Minkowski would be the Euclidean distance when *r* = 2 [23, 24]. When a set of *n* vectors is tabulated, a distance matrix that measures the difference of vector pairs would be sufficiently powered to conduct a cluster analysis for constructing dendrograms [24].

A dendrogram was constructed using Ward’s linkage method [25], an approach utilizing centroids (i.e., mean vectors) of a cluster to compute the distance between two existing clusters (*A* and *B*) where observations having the closest characteristics are grouped together. Ward’s method is based on minimizing the sum of squared errors (SSE) as follows:

2 $$I_{AB} = SSE_{AB} - \left( {SSE_A + SSE_B } \right).$$

The within and between clusters SSE values are defined as follows:

3 $$\begin{aligned} SSE_A & = \sum_{i = 1}^{n_A } {\left( {{\bf{a}}_i - {\overline{\bf{a}}}} \right)} {\prime} \left( {{\bf{a}}_i - {\overline{\bf{a}}}} \right), \\ SSE_B & = \sum_{i = 1}^{n_B } {\left( {{\bf{b}}_i - {\overline{\bf{b}}}} \right)} {\prime} \left( {{\bf{b}}_i - {\overline{\bf{b}}}} \right), \\ SSE_{AB} & = \sum_{i = 1}^{n_{AB} } {\left( {{\bf{y}}_i - {\overline{\bf{y}}}_{AB} } \right)} {\prime} \left( {{\bf{y}}_i - {\overline{\bf{y}}}_{AB} } \right), \\ \end{aligned}$$

where **a**_*i*_ is the *i*th observation of vector in cluster *A* and $\overline{\mathbf{a} }$ is the centroid of cluster *A*; **b**_*i*_ is the *i*th observation of vector in cluster *B*, and $\overline{\mathbf{b} }$ is the centroid of cluster *B*; **y**_*i*_ is the *i*th observation of vector in cluster *AB*, and ${\overline{\mathbf{y}} }_{AB}$ is the centroid of the new cluster *AB*; *SSE*_*A*_ is SSE within cluster A; *SSE*_*B*_ is SSE within cluster B; *SSE*_*AB*_ is SSE between pairs of observations in clusters A and B.

The Ward’s method calculates distances between cluster members and the centroid of a cluster, the point at which the sum of squared Euclidean distances in multivariate space between the point itself and every other point in the cluster is minimized [24]. The finalized objective equation can be expressed as follows:

4 $$I_{AB} = \frac{n_A n_B }{{n_A + n_B }}\left( {{\overline{\bf{a}}} - {\overline{\bf{b}}}} \right){\prime} \left( {{\overline{\bf{a}}} - {\overline{\bf{b}}}} \right),$$

where $\overline{\mathbf{a} }$ and $\overline{\mathbf{b} }$ are the centroids of clusters *A* and *B,* respectively.

Ward’s method provides improved linkage as compared to the single-linkage (vulnerable to produce huge clusters and singletons), complete-linkage (tendency to produce larger cluster in the largest category of clusters and the deterioration of ratio of between to total sum of squares), and average-linkage (tendency to produce singletons and worse of ratio of between to total sum of squares) methods [26].

Cluster parameters were synthesized using *Z*-standardized scores (subtracting the mean and dividing by standard deviation) and Euclidean distance, with a subsequent linkage to a matched hierarchical cluster choropleth map to visualize where clusters of populations with diabetes occur across administrative districts. The within-cluster sum of squares provides a measure of variability of observations within each cluster, such that clusters with small sum of squares values are more compact in covariate space as compared to clusters that have larger sum of squares values, relatively ‘dispersed’ across covariate values. The hierarchical clustering analysis was executed using GeoDa version 1.18 software (Center for Spatial Data Science University of Chicago, IL, USA) [27].

To understand the demographic, population, and geographic characteristics of the clusters, this study explored visualizations of the distributions between clusters in each attribute through a series of high-resolution violin plots with quartile lines, facilitated with kernel density estimation (KDE) plots, synthesized using GraphPad Prism version 8.0.1 software [28]. Most of the attributes were skewed, suggesting medians and interquartile ranges would be preferable summaries over means, motivating the use of non-parametric analyses below.

Multivariate non-parametric Kruskal–Wallis *H* test with post hoc pairwise Dunn’s tests using Bonferroni correction for multiple comparisons was performed to identify statistically significant differences between pairs of clusters. Analysis was conducted using R version 4.2.0 software [29] through the MultNonParam package [30]. A conservative threshold for *P*-values was assigned (confidence level at 99%) to define statistical significance, consistent with a previous approach [10].

## Results

### Association Between Socio-economic Indicators and Diabetes Rates by Local Districts in Malaysia

**Fig. 1:**
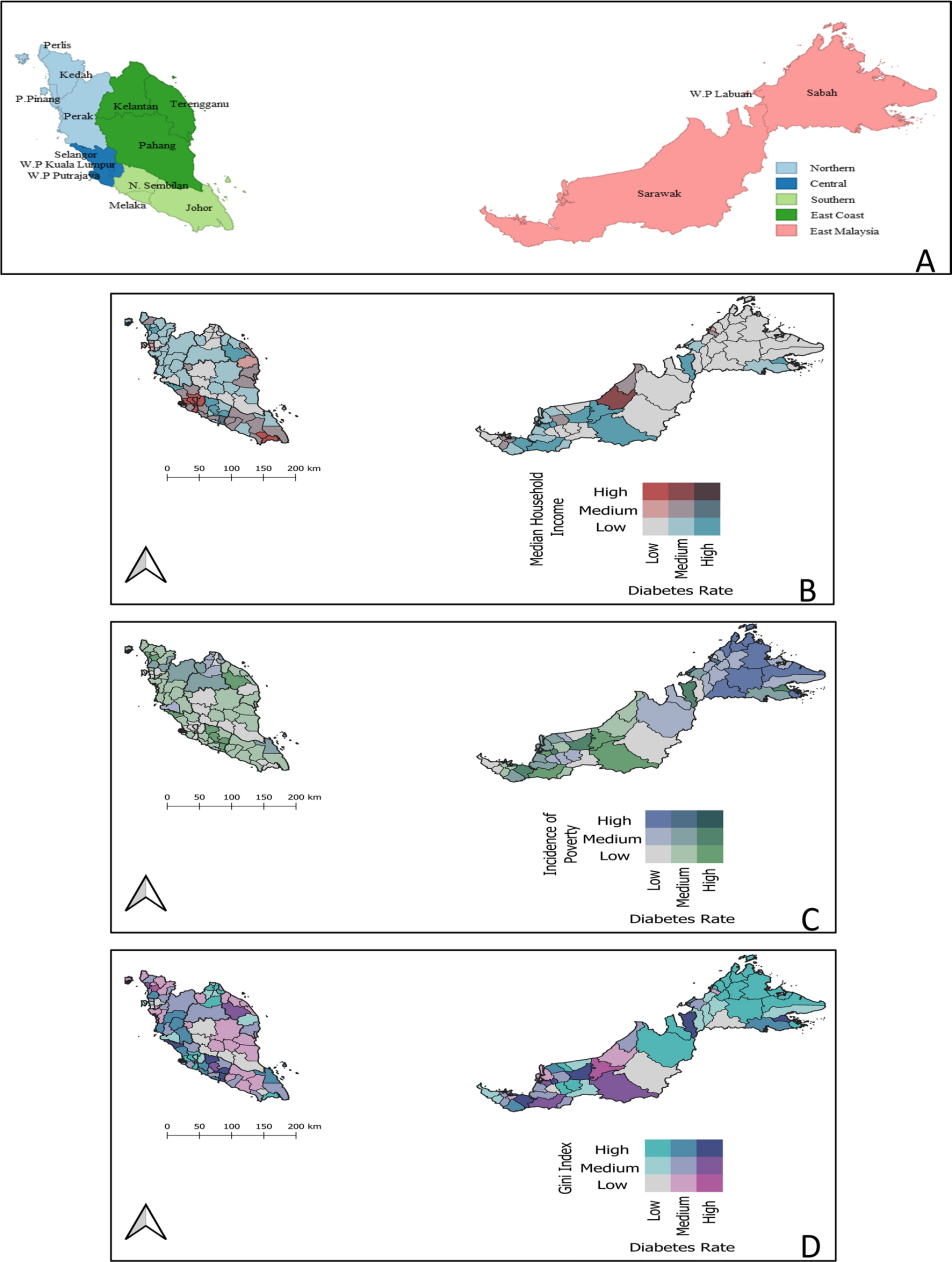
Panels of choropleths showing: **A** Base map of the states nested within regions of Malaysia; **B** Bivariate map showing associations between median household income and diabetes rates; **C** Bivariate map showing associations between incidence of poverty and diabetes rates; **D** Bivariate map showing associations between Gini coefficient and diabetes rates

Figure 1 shows a series of bivariate maps illustrating associations between socio-economic indicators (median household income, Gini coefficient, incidence of poverty) and diabetes rates by local districts in Malaysia. The maps reveal districts concentrated within the states of Negeri Sembilan, parts of Selangor, Kedah, Terengganu, Sarawak, and Sabah experiencing lower median household incomes and relatively higher diabetes rates. The remaining districts report low to medium rates of diabetes and their respective median household income values varied from low to high across different districts (Fig. 1B). Similar patterns were observed for associations between incidence of poverty and diabetes rates (Fig. 1C). Despite lower income inequalities (measured through the Gini index), diabetes rates were relatively higher in most districts across the Northern, Southern, and East Coast regions of Peninsular Malaysia. Districts with higher diabetes rates and greater income inequalities were spotted in parts of Negeri Sembilan and affluent within the Central region of Peninsular Malaysia, and parts of Sabah and Sarawak (Fig. 1D).

### Taxonomy of Area-Level Diabetes Rates Influenced by Socio-economic Inequalities

**Fig. 2:**
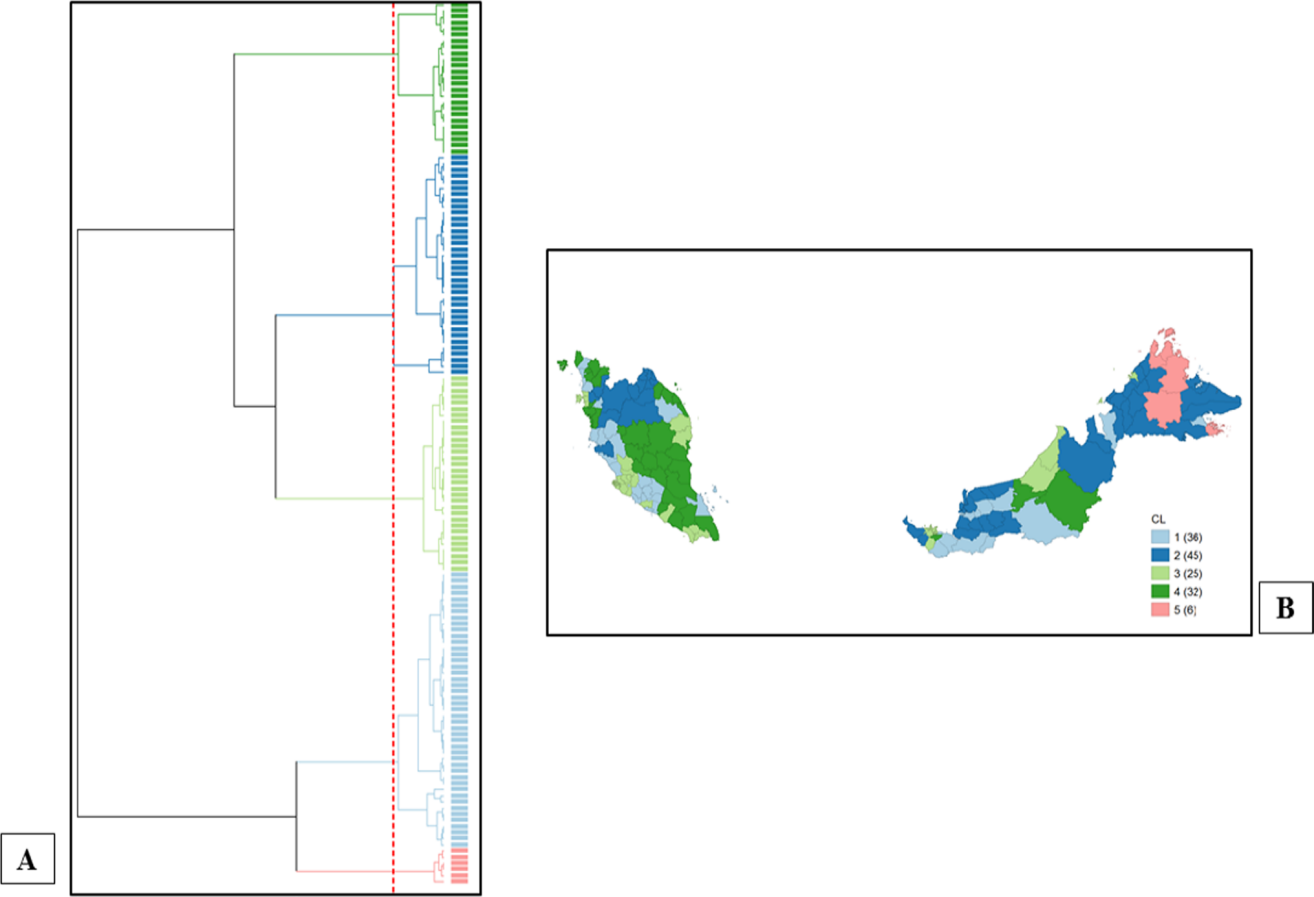
**A** Dendrogram of hierarchical cluster analysis that taxonomized diabetes rates per 100,000 population correlated with socio-economic inequalities by administrative districts in Malaysia. **B** Cluster map illustrating the geographic patterns of the clustering structures reported in the dendrogram. [Note: Dendrogram synthesized using Ward’s Linkage method with standardized (*Z*-value) transformation and Euclidean as distance function; dashed red line corresponds to cut point that yielded five clusters with statistical significance]

Figure 2A exhibits nested clusters represented by a dendrogram. Five significant clusters of different sizes were identified, each constituting different numbers of administrative districts based on concentrations of diabetes rates correlated with socio-economic inequalities. The clusters were weighted geographically and their locations were added to the hierarchical cluster map as visualized in Fig. 2B.

Clusters 2–4 were nested within larger clusters and significantly distinguishable from clusters 1 and 5 (Fig. 2A). The results highlight local patterns of diabetes rates mediated by socio-economic inequalities between clusters 1 (highest diabetes rates and median household income, narrower income inequality, and moderate incidence of poverty) and 5 (lowest diabetes rates and median household income, wider income inequality, and highest incidence of poverty) and the remaining clusters. In contrast, cluster 4 exhibits the highest diabetes rates and median household income with lower income inequalities and incidence of poverty as compared to clusters 2 and 3.

Cluster 1 contains 36 districts (average median household income MYR 4147.53; Gini coefficient 0.37; incidence of poverty 8.76%) and experienced an average diabetes rate of 2811.39 cases per 100,000 population. Collectively, these districts consist of some affluent administrative and small-business enterprises towns within rural and sub-urban areas, mostly located within the Southern, Central, and Northern regions of Malaysia. Cluster 2 contains 45 districts (average median household income MYR 3586.22; Gini coefficient 0.37; incidence of poverty 17.31%) with an average diabetes rate of 1057.48 per 100,000 population. These areas are largely rural and concentrated within the East Coast and East Malaysia regions. Cluster 3 includes 25 districts (average median household income MYR 6948; Gini coefficient 0.36; incidence of poverty 2.75%) with an average diabetes rate of 1304.46 cases per 100,000 population. These districts largely comprise urban metropolitan areas containing affluent urban and industrial towns and contributing to the nation’s economic growth. Cluster 4 consisted of 32 districts (average median household income MYR 4398.47; Gini coefficient 0.31; incidence of poverty 5.72%) and experienced an average diabetes rate of 1914.12 cases per 100,000 population. Cluster 4 consists of mixed rural areas (i.e., districts concentrated within the states of Pahang and Terengganu in the East Coast region; parts of districts within the states of Negeri Sembilan, Perlis, Kedah, and Sarawak) and some affluent developing economic towns and urban districts within the states of Johor and Pulau Pinang. Cluster 5 contains six districts (average median household income MYR 2600.67; Gini coefficient 0.43; incidence of poverty 45.72%) and was the only cluster that experienced the lowest rate of diabetes of 467.40 per 100,000 population. This cluster largely constitute rural districts concentrated in the East Malaysia region. The overall measure of fit was 0.64 (Table 1).

**Table 1:** Hierarchical Cluster Characteristics (Ward, *k* = 5)

Clusters	Cluster centers					Within-cluster sum of squares
	Administrative districts	Diabetes rates (per 100,000 population)	Median household income (MYR)	Income inequality (Gini coefficient)	Incidence of poverty (percentage)	
Cluster 1	Asajaya, Batang Padang, Hulu Terengganu, Jasin, Jelebu, Jempol, Kampar, Kapit, Kinta, Kota Setar, Kuala Muda, Kuala Pilah, Kuala Selangor, Kubang Pasu, Kunak, Lawas, Lubok Antu, Manjung (Dinding), Melaka Tengah, Meradong, Mersing, Perak Tengah, Port Dickson, Putatan, Rembau, Sabak Bernam, Sarikei, Selangau, Seremban, Serian, Sibu, Simunjan, Sri Aman, Tampin, Wilayah Persekutuan Putrajaya, Yan	2811.39	4147.53	0.37	8.76	63.90
Cluster 2	Bachok, Baling, Bau, Beaufort, Betong, Dalat, Daro, Gua Musang, Hilir Perak, Jeli, Julau, Kanowit, Keningau, Kinabatangan, Kota Belud, Kota Bharu, Kuala Kangsar, Kuala Krai, Kuala Penyu, Kulim, Lahad Datu, Limbang, Lundu, Machang, Marudi, Matu, Mukah, Nabawan, Pakan, Papar, Pasir Mas, Pasir Puteh, Penampang, Ranau, Sandakan, Saratok, Sipitang, Song, Tambunan, Tanah Merah, Tawau, Tenom, Tuaran, Tumpat, Ulu Perak	1057.48	3586.22	0.37	17.31	68.16
Cluster 3	Alor Gajah, Barat Daya, Bintulu, Dungun, Gombak, Johor Bahru, Kemaman, Klang, Kota Kinabalu, Kuala Langat, Kuching, Kulaijaya, Miri, Muar, Petaling, Pontian, Seberang Perai Tengah, Seberang Perai Utara, Seberang Perai Selatan, Sepang, Timur Laut, Ulu Langat, Ulu Selangor, Wilayah Persekutuan Labuan, Wilayah Persekutuan Kuala Lumpur	1304.46	6948	0.36	2.75	38.61
Cluster 4	Bandar Baharu, Batu Pahat, Belaga, Bentong, Bera, Besut, Cameron Highlands, Jerantut, Kerian, Kluang, Kota Tinggi, Kuala Terengganu, Kuantan, Langkawi, Larut dan Matang, Ledang, Lipis, Maran, Marang, Padang Terap, Pekan, Pendang, Perlis, Pokok Sena, Raub, Rompin, Samarahan, Segamat, Setiu, Sik, Tatau, Temerloh	1914.12	4398.47	0.31	5.72	27.72
Cluster 5	Beluran, Kota Marudu, Kudat, Pitas, Semporna, Tongod	467.40	2600.67	0.43	45.72	7.58

Cluster parameters	
Total within-cluster sum of squares	205.97
Total sum of squares	572
The between-cluster sum of squares	366.03
Ratio of between to total sum of squares	0.64

### Interpretation of Clustering Patterns Characterized by Area-Level Demographic and Population Indicators

**Fig. 3:**
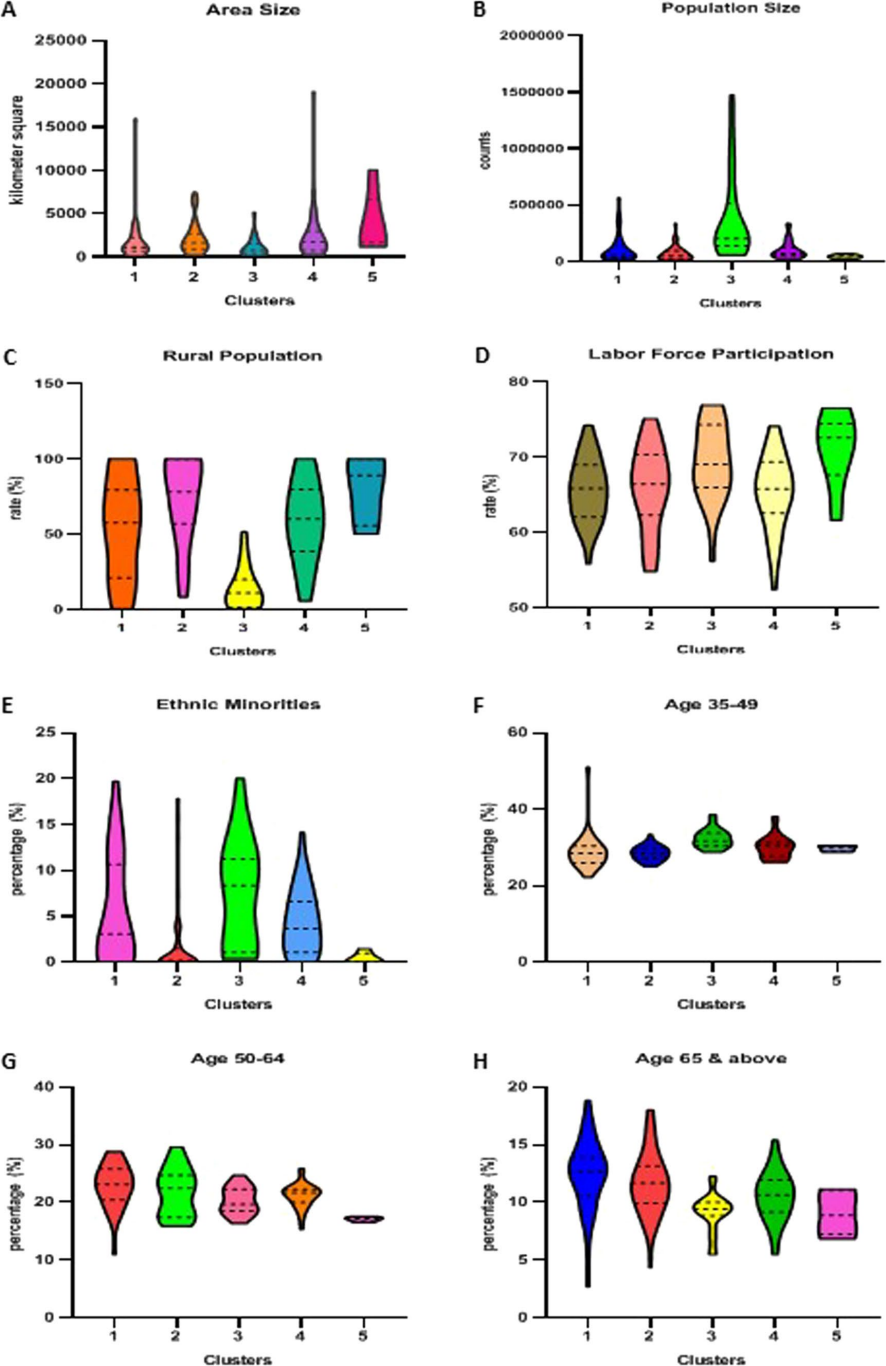
Violin plots of cluster characteristics: **A** area size in square kilometers; **B** population size; **C** percentage of population living in rural areas; **D** labor force participation rate; **E** percentage of population from Indians and others; **F** percentage of population aged 35–49 years; **G** percentage of population aged 50–64 years; **H** percentage of population aged 65 years and above

Figure 3 shows panels of violin plots characterizing how clusters of diabetes rates within administrative districts differ in area and population size, age structure, proportion of population living in rural areas, proportion of ethnic minorities, and labor force participation rates based on My Census 2020 indicators as the reference point.

It was observed that clusters varied in geographic area size (*H* statistic = 15.01, *df* = 4, *P* = 0.005; post hoc Dunn’s tests for pairwise comparisons between cluster 3 and clusters 2 and 4 were statistically significant at *P* < 0.001). Cluster 3 contained the highest population as compared to the other clusters (*H* statistic = 42.87, *df* = 4, *P* < 0.001; post hoc Dunn’s test for pairwise comparisons between cluster 3 and clusters 1, 2, 4, and 5 was statistically significant at *P* < 0.001). In a similar fashion, cluster 3 had the least proportion of the cluster population residing in areas classified as rural when compared to the other clusters (*H* statistic = 49.99, *df* = 4, *P* < 0.001; post hoc Dunn’s tests for pairwise comparisons that differed between cluster 3 and clusters 1, 2, 4, and 5 was statistically significant at *P* < 0.001). It was evident that clusters varied in terms of their respective population’s labor participation (*H* statistic = 15.09, *df* = 4, *P* < 0.005). In pairwise comparisons, cluster 3 experienced higher labor force participation rate than cluster 1, but lower in comparison to cluster 5 (post hoc Dunn’s test significant at *P* = 0.005). There also appeared to be differences in the proportion of ethnic minorities (*H* statistic = 32.81, *df* = 4, *P* < 0.001; post hoc Dunn’s test for pairwise comparison between cluster 2 and clusters 1, 3, and 4, and clusters 3 and 5 were statistically significant at *P* < 0.001) (Table 2).

**Table 2:** Multivariate non-parametric Kruskal–Wallis *H* test with post hoc multiple comparisons

Characteristics	Median	*H* statistic	*P*-value	Post hoc Dunn’s test for multiple comparisons									
				CL 1–2	CL 1–3	CL 1–4	CL 1–5	CL 2–3	CL 2–4	CL 2–5	CL 3–4	CL 3–5	CL 4–5
*Area size*													
Cluster 1	1076.5	15.01	0.005					***			***		
Cluster 2	1600												
Cluster 3	715												
Cluster 4	1733												
Cluster 5	1668												
*Population size*													
Cluster 1	55,450	42.87	< 0.001		***			***			***	***	
Cluster 2	47,200												
Cluster 3	198,600												
Cluster 4	65,950												
Cluster 5	40,250												
*Rural population*													
Cluster 1	57.5	49.99	< 0.001		***			***			***	***	
Cluster 2	78.2												
Cluster 3	10.75												
Cluster 4	60												
Cluster 5	89.05												
*Labor force participation*													
Cluster 1	65.8	15.09	0.005		**			**			**	**	
Cluster 2	66.4												
Cluster 3	69.0												
Cluster 4	65.75												
Cluster 5	72.6												
*Ethnic minorities*													
Cluster 1	3.07	32.81	< 0.001	**				**	**			**	
Cluster 2	0.16												
Cluster 3	8.36												
Cluster 4	3.63												
Cluster 5	0												
*Age 35–49*													
Cluster 1	28.59	36.33	< 0.001		**			**			**		
Cluster 2	28.57												
Cluster 3	31.70												
Cluster 4	30.47												
Cluster 5	29.97												
*Age 50–64*													
Cluster 1	23.12	22.35	< 0.001		***		***			***			***
Cluster 2	22.48												
Cluster 3	19.68												
Cluster 4	21.69												
Cluster 5	17.17												
*Age 65 and above*													
Cluster 1	12.64	31.54	< 0.001		***	***	***	***					
Cluster 2	11.68												
Cluster 3	9.41												
Cluster 4	10.63												
Cluster 5	8.89												

Degrees of freedom (*df* = 4) for all parameters

**Denotes *P* < 0.005

***Denotes *P* < 0.001

Differences were observed in the proportion of population aged 35–49 years (*H* statistic = 36.33, *df* = 4, *P* < 0.001; post hoc Dunn’s test for pairwise comparison significant between cluster 3 and clusters 1, 2, and 4 at *P* < 0.001), 50–64 years (*H* statistics = 22.34, *df* = 4, *P* < 0.001; post hoc Dunn’s test for pairwise comparison significant between cluster 1 and 3, and cluster 5 and clusters 1, 2, and 4 at *P* < 0.001), and aged 65 and above (*H* statistics = 31.54, *df* = 4, *P* < 0.001; post hoc Dunn’s test for pairwise comparison significant between cluster 2 and 3, and cluster 1 and clusters 3, 4, and 5 at *P* < 0.001). Clusters 3 and 5 had higher proportions of younger residents (ages 35–49) with diabetes, whereas the proportion of diabetes in older aged people (aged 50 and above) was higher in areas within clusters 1, 2, and 4, respectively (Table 2).

## Discussion

### Core Summary Findings

The distributional patterns revealed in the choropleth maps reveal the highest proportions of persons afflicted with diabetes residing within districts concentrated in the states of Negeri Sembilan, parts of Selangor, Kedah, Terengganu, Sarawak, and Sabah, whereas other areas experience low to moderate rates of diabetes. This distributional pattern coexisted with different socio-economic inequalities across administrative districts in Malaysia. To explore potential associations, a hierarchical clustering technique was employed to classify regions by both diabetes rates and by socio-economic indicators. Our analyses identified five statistically distinct clusters containing different groupings of administrative districts with varying proportions of people with diabetes, nested within distinct socio-economic inequalities between areas.

Clusters 1 and 5 showed contrasting pattern of diabetes rates, cluster 1 had the highest diabetes rates, whereas cluster 5 had the lowest. Administrative districts in cluster 1 were mostly sub-urban townships with higher median household income, reduced income inequalities, and reduced incidence of poverty as compared to cluster 5 which consisted of highly concentrated rural areas having the lowest median household income, wider income inequality, and the highest incidence of poverty. These differences are summarized in the distributional characteristics of clusters as exhibited in Fig. 3, with cluster 5 being highly rural, having greater labor force participation most likely within the agriculture, fisheries, and forestry sector that structured population’s living environment, lifestyle practices, and dietary patterns distinct from urban societies. The relatively lower rates of diabetes in rural areas could be influenced by local variations in health seeking behaviors among local communities or barriers to access necessary health services when needed, each negating timely diagnosis of diabetes. In contrast, mixed areas between urban and rural (e.g., clusters 1, 2, and 4) and highly urbanized areas (e.g., cluster 3) have seen greater burden of diabetes within lower socio-economic inequalities.

### Comparisons with Existing Literature

Previous research employed partitioning clustering (e.g., *k*-means clustering) to identify clusters that group people with diabetes according to individuals’ internal exposomes [31–34]. Such works comprise the field of diabetology, concluding biological, physiological, genetic, and demographic attributes as risk factors for diabetes in adults. Although these areas of research are crucial to accelerate clinical driven interventions, they focus on individual-level factors and are not directly set to inform community-level public health policies for health outcomes control.

From a public health perspective, researchers have used *k*-means clustering with linkage to local geographical areas to understand how well proximal determinants of health correspond to local patterns of persons with better or worse health outcomes [2]. These researchers concluded that worse health outcomes occurred in North England associated with local measures of social deprivation. Another study in the United Kingdom [10] found consistent points made by the prior study [2], but with a different clustering approach taken. That study applied hierarchical clustering methods using distal determinants of health [10], an approach providing area-level aggregate deprivation scores, confirming that ecological indicators substantially aided in identifying areas of worse health outcomes, e.g., North England, as previously reported [2].

### How This Study Differed from Previous Research?

In a multi-country cluster analysis work [32], researchers observed that the proportion of people with diabetes in each cluster was inconsistent across the countries, providing an assumption that the characteristics of individuals with diabetes in the population do not distribute equally and that risk factors may vary according to differing living circumstances of local populations. Such variations can pose difficulties for public health advocates to propose general interventions to effectively control health problems across all neighborhoods. The current study has advanced newer approaches for significant methodological implications that could accelerate the planning, implementation, and evaluation of local public health policies relating to variations in both local prevalence of diabetes and local socio-economic indicators.

#### Methodological Implications

The proposed use of hierarchical clustering in this study provides more meaningful interpretations for local public health as compared to the partitioning clustering approaches used in previous studies. The nature of hierarchical clustering is that it only requires a similarity measure of area characteristics that accelerate the risk of disease, without having to define the initial number of clusters or centers that partitioning clustering algorithm requires prior to execution. This allows hierarchical clustering to synthesize more meaningful and subjective division of clusters of co-incident diabetes rates and socio-economic variables from the field as compared to a supervised approach that partitions clustering to yield only the exact ‘pre-determined’* k* number of clusters without ‘thinking’ the characteristics or risk factors that pool together to determine areal level disease burden.

Following baseline evidence [2], this study further advanced a methodological application of hierarchical clustering with Ward’s linkage method to local administrative districts in Malaysia, a statistical neighbors’ approach that is strongly capable to identify comparable local administrative areas, a substantial limitation that was acknowledged in a previous work [10]. Ward’s linkage method offsets the limitation of a complete-linkage approach that often will produce larger clusters in the largest category of clusters. The current study’s overall index produced the ratio of between to total sum of squares of 0.64, a variability metric of compactness of observations within the clusters which confidently allows epidemiologists to interpret that the percentage of district-level diabetes rates that correctly clustered on the influence of distal determinants of health was approximately 64%, a reliable value for local public health authorities to execute areal-specific interventions in line with population’s risk of diabetes in that area.

#### Implications for Public Health Policies and Interventions

By setting a methodological framework to critically understand ‘a problem nested within a problem’ from the Asian continent, we illustrate how the burden of diabetes in Malaysia should be appraised as a ‘local area problem’ requiring local public health authorities’ tailored interventions. The clusters reveal distributional patterns of diabetes hotspots, and identify areas containing similar values of upstream social determinants of health. This work propose that the approach used offers guidance to target interventions to local areas as a feasible and cost-effective approach, since successful interventions or policies implemented in one local public health authority might transfer easily to other areas that share similar risk factors or exposures within the same cluster.

#### Future Directions

The findings of this study provide local insights countrywide, covering areas with different socio-economic inequalities and demography within the rural, sub-urban, and urban regions of the country based on the hierarchical clustering algorithm that was linked via a distance metric, using the Ward’s linkage method. As an advancement, an algorithm for Ward’s linkage to be complemented with a geographical weighted matrix to identify local areas with comparable disease burden for linking geographically nearby neighbors is proposed for future works. With rapid urbanization and industrialization, especially in low- and middle-income countries (LMICs), the population’s internal migration patterns may accelerate with changing socio-economic opportunities. Such circumstances can catalyze fast growing urban areas or cities, structured with advanced township planning, nested within urban built-environments that impose lifestyle changes resulting in people being susceptible to greater risks of non-communicable diseases. This study offers an opportunity for within-city future work where analysts could extend the statistical approach applied in this work to generalize Ward’s method for use with Manhattan, instead of Euclidean, distances when populations are concentrated within buildings or cities blocks [24], in order to examine localized clustering of health problems within urbanized cities.

The use of choropleth maps provides valuable insights at the areal level; such maps generated allow epidemiologists to better understand potential hot spots of local area disease clusters for public health policy interventions. Such cartographic approaches are important since secondary data sources often are officially validated by experts in the field (e.g., diabetes disease registries that capture diabetes cases as diagnosed by medical experts, as used in this study). In contrast, collaborative mapping requires interviewed individuals to draw or create their own maps. Such individual-level maps could be synthesized from crowdsourcing projects, such as OpenStreetMap [35] whereby communities could volunteer to upload local survey data (e.g., self-reported geolocated diabetes of individuals, aerial photographs of living settlements) as open-source data allowing epidemiologists to understand local area disease burden. Collaborative mapping or crowdsourced mapping projects could provide additional individual-level insights, but they are not validated by experts in the field, require additional effort to collect, and may be subject to recall or response bias. Moreover, since public health policies typically are executed at aggregate administrative levels and not individualized, choropleth mapping approaches were more suitable to the goals of this study. Collaborative mapping was beyond the scope of the original data-collection exercise in this study; however, future research works could undertake such mapping strategies in order to explore individual-level drivers of detected hot spots.

### Study Limitations

It should be acknowledged that areal-level risk factors and exposures change over time; however, the data used in the current study were not refined enough to explore temporally changing influences of socio-economic inequalities and trends of diabetes rates. It is recommended for future research to examine time trends and stability of clusters over time and space. Clustering techniques can be sensitive to minor changes in spatial data, and depending on the distance linkage and measures used, hence, results should be interpreted with caution [36]. Although such sensitivities cannot be eliminated, this study tried to minimize potential impacts on conclusions by adopting conservative *P*-values (a stringent alpha) for identifying statistically significant and stable branches of trees in the synthesized dendrogram.

The current study notes that education levels may not be relevant to socio-economic inequalities measurement [37]. Literacy has emerged as a measure of education quality, being more reflective to socio-economic inequalities in ethnic minorities and low-income communities [38, 39]. The measure has been directly attributed to health seeking behaviors and healthy lifestyle practices [40]. Given the limitations of no available data relating to literacy in the current study, it is recommended for future studies to expand the examination to include literacy as a covariate within clustering analysis.

This study recommends care in interpreting the influence of income inequality (as measured by the Gini coefficient) with diabetes rates. The accuracy of the Gini coefficient is highly dependent on reliable Gross Domestic Product (GDP) and income data. It should be noted that sub-urban or rural districts with varying demographics may be highly affluent with unregistered or informal economies that do not contribute to areal-level GDP growth; these areas may have different income distributions which nevertheless may yield identical Gini coefficient values as other areas and subsequently overstate true income inequalities in these districts [41, 42]. Such circumstances may influence the distribution patterns of diabetes rates as visualized in the bivariate choropleth maps.

## Conclusion

The hierarchical clustering analysis yielded five statistically distinct areal-level clusters with varying socio-economic, demographic, and geographic characteristics across Malaysia. The approach and results in this study have provided information to help target locally tailored interventions to areas with similar characteristics (i.e., within the same cluster) in order to help control the diabetes epidemic at the local level. Although such an approach offers an opportunity to bring local focus to local aspects of the uncontrolled diabetes problem in Malaysia, any direct causal interpretations of the results relating to individual-level risk should be cautioned due to the sensitiveness of the algorithms to small perturbations of spatial data and due to the ecological nature of the study data.

### Supplementary Information

Below is the link to the electronic supplementary material.

Supplementary file 1 (XLSX 18 KB)

## Data Availability

The data that support the findings of this study are available from the Ministry of Higher Education Malaysia, the Ministry of Health Malaysia, and the Department of Statistics Malaysia, but restrictions apply to the availability of these data, which were used under license and approval for the current study, and so are not publicly available. Data are, however, available from the authors upon reasonable request and with permission of the Ministry of Higher Education Malaysia, the Ministry of Health Malaysia, and the Department of Statistics Malaysia.

## Abbreviations

*df*: Degrees of freedom
GDP: Gross domestic product
KDE: Kernel density estimation
LMIC: Low- and middle-income countries
NDR: National diabetes registry
QGIS: Quantum GIS
SSE: Sum squared errors
USA: United States of America
